# Role of phytobiotics in relieving the impacts of *Aeromonas hydrophila* infection on aquatic animals: A mini-review

**DOI:** 10.3389/fvets.2022.1023784

**Published:** 2022-10-06

**Authors:** Zulhisyam Abdul Kari, Wendy Wee, Suniza Anis Mohamad Sukri, Hasnita Che Harun, Mohd Farhan Hanif Reduan, Martina Irwan Khoo, Hien Van Doan, Khang Wen Goh, Lee Seong Wei

**Affiliations:** ^1^Department of Agricultural Sciences, Faculty of Agro-Based Industry, Universiti Malaysia Kelantan, Jeli, Kelantan, Malaysia; ^2^Center of Fundamental and Continuing Education, Universiti Malaysia Terengganu, Kuala Nerus, Terengganu, Malaysia; ^3^Department of Paraclinical Study, Faculty of Veterinary Medicine, Universiti Malaysia Kelantan, Kota Bharu, Kelantan, Malaysia; ^4^Department of Chemical Pathology, School of Medical Sciences, Universiti Sains Malaysia, Kota Bharu, Kelantan, Malaysia; ^5^Department of Animal and Aquatic Sciences, Faculty of Agriculture, Chiang Mai University, Chiang Mai, Thailand; ^6^Science and Technology Research Institute, Chiang Mai University, Chiang Mai, Thailand; ^7^Faculty of Data Science and Information Technology, INTI International University, Nilai, Malaysia

**Keywords:** motile aeromonad septicemia (MAS), plant extract, anti-bacterial activity, innate immunity, disease resistance, synergistic

## Abstract

*Aeromonas hydrophila* is a ubiquitous bacterium with various hosts that causes mass mortality in farm-raised fish species and significant economic losses. The current antibiotic treatment is ineffective in controlling this bacterium infection in aquaculture species. Therefore, an evaluation of potential phytobiotics is needed to find an alternative antimicrobial agent to reduce the over-reliance on antibiotics in aquaculture and safeguard public and environmental health. Furthermore, the rise in antibiotic resistance cases among pathogenic bacteria indicates an urgent need for new fish and shellfish health management solutions. In this context, phytobiotics applications in aquaculture can be defined as any medicinal plant-based antimicrobial agent used in fish and shellfish health management. This review will focus on the impacts of Motile Aeromonas Septicemia (MAS) due to *A. hydrophila* in aquaculture, the potential of phytobiotics in enhancing the tolerance of aquaculture species against MAS and the combination of phytobiotics with other antimicrobial and therapeutic agents against MAS.

## Introduction

The aquaculture industry is essential in sustaining more than 1 billion population worldwide who depends on fish as their primary source of protein (1, 2). This fast-growing industry (3, 4) recorded an all-time high production, with a total live weight of 114.5 million tons (5). As aquaculture intensifies, diseases have become a major constraint for the industry, i.e., Motile Aeromonad Septicemia (MAS) caused by *Aeromonas hydrophila*. *A. hydrophila* can be found in various environments such as marine, freshwater, brackish water, water supplies, and incredibly abundant during the warmer seasons (6). The hosts of *A. hydrophila* are vast, ranging from freshwater to marine aquatic species (7). This bacterium was also reported to be coinfect with epizootic ulcerative syndrome (EUS) (8). Thus, this highly virulent microorganism may cause mass mortality of aquaculture species on farm sites (9).

Aquatic animals infected by MAS exhibit symptoms such as cloacal hemorrhage (10), ascites (11), gastroenteritic hemorrhage (12), and septicemia ulceration on the skin (13). Furthermore, the *A. hydrophila* strain will determine the appearance of the symptoms in the infected fish (14). Serine protease (ser), cytotoxins and aerolysin (aer) are the common virulence factors that contribute to *A. hydrophila* pathogenicity (15). In addition, lipase (lip), cytotonic enterotoxins (act, alt, ast), polar flagella (fla), DNases (exu), type III secretion system (ascV), cholesterol acyltransferase (gcaT), and elastase (ahyB) have been associated with *A. hydrophila* pathogenicity (16–18). These virulence factors, in combination with other factors, will lead to the pathogenicity of *A. hydrophila* (19).

Traditionally, fish farmers use antibiotics as prevention and treatment for aquaculture species health management. However, the misuse and overexploitation of antibiotics have led to increasing antibiotic resistance cases among pathogenic bacteria from aquaculture sites (20). For example, *A. hydrophila*, isolated from Nile tilapia, *Oreochromis niloticus*, was resistant to ampicillin and amoxicillin (21). Therefore, fish farmers have no choice but to increase antibiotic dosages to treat diseases in aquaculture. A study suggests a combination of two different antibiotics, thiamphenicol, and florfenicol, were effective at lower dosages against *A. hydrophila* infection in Nile tilapia (22). Consequently, studies are ongoing to find alternative treatments such as vaccination programs (23) and phytobiotics applications (24–28). Various studies have revealed the potential of phytobiotics as an alternative to antibiotics, thus, minimizing antibiotics usage in aquaculture. Phytobiotics contain bioactive compounds such as alkaloids, sterol, flavonoids, saponins, and tannins that possess bactericidal properties and stimulatory effects (29). For example, miswak, and *Salvadora persica*, contain broad spectrum bactericidal compounds such as benzyl isothiocyanate, salvadourea, salvadorine (29), and vitamin C that can promote healing and tissue repair (30).

This review summarized the impacts of MAS caused by *A. hydrophila* in aquaculture, phytobiotics preparation for aquaculture uses, the antibacterial activity of medicinal herbs in aquaculture, phytobiotics-activated innate immunity in aquaculture species, phytobiotics enhanced tolerance of aquaculture species toward MAS caused by *A. hydrophila* and phytobiotics in combination with other antimicrobial and therapeutic agents against MAS caused by *A. hydrophila*.

## Impacts of MAS caused by *A. hydrophila* in aquaculture

Disease outbreak is a major constraint to the growth of the aquaculture industry. One of the sources of diseases is *A. hydrophila*, an opportunistic pathogen associated with secondary infection (31) and outbreaks (see Table 1). Fish infected with MAS will exhibit symptoms such as lose appetite, skin ulcerations, pale gills, swollen abdomen, and abnormal swimming pattern. Antibiotics such as oxytetracycline and terramycin were commonly used for treatment. *A. hydrophila* can cause MAS that has detrimental impacts *o*n aquaculture, such as devastating fish farms, causing economic losses, and pose a threat to public health and environmental safety. This disease was first reported by Llobrera and Gacutan (32) in snakehead, catfish, carp and goby in Laguna de Bay, Philippines, where the infected species exhibited lesions and necrotic ulcers. Many studies reported that MAS was responsible for the mass mortality of aquaculture species. For instance, a mass mortality of Channel catfish, *Ictalurus punctatus*, in commercial fish farms in the Southeastern USA (31). Furthermore, the total loss of this outbreak in commercial fish farms amounted to millions of USD, leading to numerous fish farm closures (33).

**Table 1:** Impacts of MAS in aquaculture.

**Aquatic species**	**Impacts**	**Prophylactic measures**	**Location**	**References**
Freshwater Murray cod, *Maccullochella peelii*	Tail rot disease, mass mortality	Cephalosporin, chloromycetin, glycopeptides, macrolides, nitrofuran and penicillin drugs	Shanghai, China	(34)
Channel catfish, *Ictalurus punctatus*	Motile Aeromonad Septicemia (MAS)	Probiotic, vaccination	Southeastern USA	(31)
Catfish, *Clarias gariepinus*	80–100% mortality in 2 weeks	Maintain good water quality for prevention of MAS infection	Surabaya, Indonesia	(35)
Freshwater cultured whiteleg shrimp, *Litopenaeus vannamei*	Emerging causative agent; mass mortality of shrimp	Combination florfenicol and *Punica granatum*	Fengxian, Shanghai, China	(36)
Nile, tilapia *Oreochromis niloticus*	Mass mortality	Not mentioned	São Paulo, Brazil	(37)
Nile tilapia, *O. niloticus*	High mortality	Maintain good water quality for prevention of MAS infection	Egypt	(38)
Nile tilapia, *O. niloticus*	35–50% mortality; *A. hydrophila* carrying the antibiotic resistance gene	Ciprofloxacin	Egypt	(21)

The MAS is also reported to alter the appearance of an aquaculture species, contributing to the losses in market value. For example, Dierckens et al. (39) claimed that the Fairy shrimp, *Branchinecta gigas* (Lynch), infected with MAS appeared black, causing a substantial price drop. Furthermore, the risk of mass mortality among aquaculture species due to this disease forced fish farmers to harvest and sell their products in the market immediately to reduce losses. Moreover, disease control *via* antibiotics administration without adequately assessing the effectiveness of the treatments contributes to the infiltration of residues in the environment and microflora breakdown in the area (40), besides posing a threat to public health and the environment.

## Phytobiotics vs. commercially developed vaccines against MAS

Fish vaccination was developed more than 50 years ago (41) and reported to be effective in disease control. The vaccines in aquaculture can be administrated *via* immersion or intraperitoneal injection. In addition, live vaccines were reported to be highly effective in stimulating vigorous antibody activity in fish when administered orally or by immersion. An ideal vaccine is safe for the fish, environmentally friendly, cost-effective, user friendly, and highly effective with little or no side effects (41). In a recent study, fish vaccination has been improved by dissolving microneedle patches. Yun et al. (42) developed this novel vaccination administration to prevent *A. hydrophila* infection in fish. Dissolving microneedle patches were more effective and can be an alternative to injection in fish vaccination. Currently, the commercial vaccine (Alphaject Panga 2) is used against MAS, in stripped catfish farming in Vietnam (41). However, there are several issues with using the vaccine in aquaculture. High cost and labor intensive are two major factors to be considered by the fish farmer where these factors will hinder vaccines applied extensively in aquaculture.

On the other hand, phytobiotics are plants or plant-based bioactive compounds beneficial for farmers, animals and humans. Examples of phytobiotics are essential oil, legumes, herbs, fruits and vegetables, alkaloids, carotenoids, and phenolic compounds (43, 44). These phytobiotics are widely used in aquaculture feed additives to enhance the immune system and protect aquatic animals against MAS. Since phytobiotics are abundant and inexpensive, fish farmers utilized this treatment as a vaccine replacement in aquaculture health management.

## Phytobiotics preparation for aquaculture uses

There are several methods to prepare phytobiotics for aquaculture uses, such as aqueous extracts, methanol extract, and powder form. Methanol is a universal solvent used to extract polar compounds, especially in plants (45), whereas aqueous extracts derive non-polar compounds in phytobiotics. Several studies utilize methanol as a polar aqueous solvent for non-polar extraction in phytobiotics preparation. For example, Wei et al. (46) and Zhou et al. (36) compared the efficacy of bacterial inhibition between methanolic and aqueous extracts. Most studies used methanol in phytobiotics preparation since this solvent was found effectively derives all the properties in the sample (45), Sheikhlar et al. (47), Lee et al. (20), Sheikhlar et al. (48), Bao et al. (49), and Rashmeei et al. (50). The extracts were then used as feed additives in the feeding trials.

Phytobiotics can also be prepared in powder form. For example, Thanikachalam et al. (51) prepared dried garlic peel powder before being incorporated into fish feed for a feeding trial. Similarly, phytobiotics are prepared as feed additives using Rosemary leaf (52), *Psidium guajava* L. leaf (53, 54), and Spirulina (*Arthrospira platensis*) (55). Furthermore, some phytobiotics can be commercially available such as tapioca (56), curcumin (57), origanum essential oil (38), and thyme, red thyme and pepper rosemary essential oil (58). Recently, studies have utilized the polysaccharide derived from *Pistacia vera* hulls (59) or fermentation in preparing phytobiotics (60).

## Antibacterial activity of medicinal herbs in aquaculture

Despite the rapid action of antibiotics in controlling a disease outbreak, the residues can disrupt the natural microflora by seeping into the surrounding sediment and water bodies (61). Furthermore, antibiotics such as oxolinic acid, flumequine, oxytetracycline, and sulfadiazine are stable for up to 3 months in soil (62). Thus, there is a dire need for an alternative antimicrobial agent for aquaculture uses.

Medicinal herbs are extensively used as a treatment for human diseases. The whole plant or part of the herbaceous plant, such as twigs, roots, stem, flower, and fruit, are subjected to the extraction process for bioactive compound derivation (63). Bioactive compounds include terpenoids, tannins, alkaloids, and flavonoids, which possess antibacterial properties (64). Furthermore, numerous studies have revealed the potential of medicinal herbs for aquaculture uses; thus, researchers have developed new and improved approaches for antibacterial discoveries from plants. For instance, antibacterial properties were characterized in polysaccharides derived from macroalgae, *Chaetomorpha aerea* (65) and nanoscale silver particles of butter fruit, *Persea americana* (66). These studies highlighted future research prospects for plant-based polysaccharide-derived compounds and nano-synthetic substances.

Based on the literature, many medicinal herbs were reported to demonstrate antibacterial properties against *A. hydrophila*. For instance, *Murraya koenigii, Pandanus odoratissimus* (46), *Colocasia esculenta* (67), and *Euphorbia hirta* (48) inhibited the growth of *A. hydrophila* (Table 2). These medicinal herbs contain bioactive compounds, such as carbazole alkaloids (68), phenolic compounds (69), polypeptides (70), and alkaloids (71), that was responsible for the antibacterial activities. Moreover, recent studies showed that pomegranate, *Punica granatum* (Peel), *Prunus mume* (fruit), *Fructus toosendan* (fruit), *Artemisia argyi* (leaves), *Polygonum aviculare* (leaves), *Cephalanoplos segetum* (leaves), and *Artemisia capillaries* (leaves) demonstrated the ability to inhibit *A. hydrophila* activities (36). Similarly, *Piper betle, Piper sarmentosum*, and *Piper nigrum* demonstrated inhibitory activities, as reported by Anjur et al. (72).

**Table 2:** Antibacterial activities of medicinal herbs in aquaculture.

**Medicinal herbs**	**Pathogen**	**Measurement method**	**References**
*Murraya koenigii, Pandanus odoratissimus*	*Aeromonas hydrophila*	Well diffusion	(46)
*Colocasia esculenta*	*Vibrio* spp.	Disk diffusion	(67)
Ethanol extract of *Asparagopsis taxiformis*	*Vibrio alginolyticus, Vibrio vulnificus, Aeromonas salmonicida*	Antimicrobial test	(73)
Methanol extract of *Euphorbia hirta* (Aerial part)	*A. hydrophila*	Disk diffusion	(48)
Pomegranate, *Punica granatum* (Peel), *Prunus mume* (fruit), *Fructus toosendan* (fruit), *Artemisia argyi* (leaves), *Polygonum aviculare* (leaves), *Cephalanoplos segetum* (leaves), and *Artemisia capillaries* (leaves)	*A. hydrophila*	Macro broth dilution	(36)
*Scutellaria baicalensis* (root)	*A. hydrophila, Edwardsiella tarda, Vibrio alginolyticus, Vibrio harveyi*	Antimicrobial test, anti-inflammation assay	(74)
Crude extract or polysaccharides of macroalgae, *Chaetomorpha aerea*	*Listonella anguillarum, V. harveyi, V. parahaemolyticus* and *Photobacterium damselae*.	Antimicrobial test	(65)
Nanoscale silver particles butter fruit, *Persea americana*	*Providencia vermicola*	Antimicrobial test	(66)
*Piper betle, Piper sarmentosum*, and *Piper nigrum* crude aqueous extract	*A. hydrophila*	Disc diffusion and resazurin microdilution assays	(72)

## Phytobiotics activated the innate immunity of aquaculture species

Antibiotics are essential for aquaculture species health management (40, 75, 76). Nevertheless, antibiotic action is not limited to the target bacteria but also other microorganisms in aquatic animals and the environment (77). Moreover, antibiotic residues in the environment contribute to the development of resistance genes in the microorganisms (78). Vivekanandhan et al. (79) reported that almost 99% of *A. hydrophila* isolated from seafood (fish and prawn) in the wet market of South India were resistant to novobiocin, bacitracin, rifampicin, and methicillin. Meanwhile, De Silva et al. (80) have revealed that all *A. hydrophila* isolated from Yesso scallop, *Patinopecten yessoensis* were resistant to ampicillin, colistin, vancomycin, and cephalothin. Therefore, phytobiotics offer an alternative solution in aquaculture species health management to overcome antibiotic resistance.

Phytobiotics can also activate the innate immunity of aquaculture species to stimulate disease resistance. Many studies have proven the potential of phytobiotics as an alternative antimicrobial agent for aquaculture uses. For instance, *Boswellia serrata* resin extract was reported can be phytobiotic to enhance the immune system of Nile tilapia and *Oreochromis niloticus* against *Staphylococcus aureus* infection (81). In the study, *B. serrata* resin extract can activate the innate immune system of *O. niloticus via* the immune response assay, disease resistance assay and growth performance experiment. Similar findings were reported on *Spophora flavescens* (82), peppermint (*Mentha piperita*) (83), Aloe vera powder (84), *Spirulina platensis* (85), citrus lemon peel essential oil (86). The mentioned phytobiotics can improve aquaculture species' growth performance and activate their innate immune system against diseases.

Phytobiotics also stimulate mucus production on fish skin, which acts as a non-specific immunity. Fish skin mucus serves as a primary physical defense against various pathogens. A recent study showed that feeding Siberian sturgeon (*Acipenser baerii*) with barbery fruit extract for 56 days caused a remarkable improvement in the skin mucus bactericidal activity against *A. hydrophila*, where the bacterial inhibition zone diameter is higher compared to the fish fed with unsupplemented diet (control group) (87). Another phytobiotics mechanism is through immune cells improvement and macrophages activation, as reported by Chen et al. (88), where *Salvia miltiorrhiza* polysaccharide exhibited the ability to modulate the disease resistance of sturgeon against *A. hydrophila* infection. In addition, phytobiotics from plant secondary metabolites such as saponin, essential oils, phenolic compounds, polysaccharides, and polypeptides demonstrated the potential to treat the bacterial infection with low toxicity (89). The phytobiotics can also act as active site modulators, enzymes as catalytic sites, receptors and proteins for disease treatments (90).

## Phytobiotics enhanced aquaculture species tolerance against MAS

Existing studies indicated that fish farmers utilize antibiotics, chemicals, phytobiotics, probiotics, prebiotics, yeast extract, vaccine, and disinfectants to control MAS caused by *Aeromonas hydrophila* in commercial farms (91). Nonetheless, these treatments have been proven unsustainable in preventing MAS caused by *A. hydrophila*. This catastrophic bacterial disease is responsible for the mass mortality of aquaculture species and significant economic losses. Nevertheless, excessive antibiotic usage will increase antibiotic resistance among pathogenic bacteria, leading to its inefficacy in aquaculture disease control. Therefore, fish farmers must be given more options to use other antimicrobial agents instead of over-relying on antibiotics. Recent studies showed promising findings on phytobiotics in controlling *A. hydrophila* infection in aquaculture species. The phytobiotics were used as a feed additive given together with feed to aquaculture species for some time can activate and enhance the immune system of aquaculture species against *A. hydrophila*. For example, Zhang et al. (92) revealed that 1–2% of *Flos populi* extract incorporated in feed and given to Goldfish (*Carassius auratus*) for 45 consecutive days could enhance medicated fish growth, antioxidative status, non-specific immunity, and disease resistance to *A. hydrophila* infection (Table 3). Many other studies have shown a similar trend of findings in the literature. Therefore, there is no doubt phytobiotics can enhance the tolerance of aquaculture species against Motile Aeromonas Septicemia.

**Table 3:** Medicinal herbs used to mitigate *A. hydrophila* impacts on aquatic animals.

**Species**	**Medicinal herbs**	**Dose**	**Duration**	**References**
Rohu, *Labeo rohita*	Garlic, *Allium sativum*	1–10 g per kg of fish	10 days	(93)
Nile tilapia, *O. niloticus*	*Psidium guajava* (Dried leaf powder)	1 feed: 4–24 *Psidium guajava* (dried leaf powder)	10 days	(54)
Nile tilapia, *O. niloticus*	Chinese medicinal herbs, *Astragalus membranaceus* and *Lonicera japonica*	*Astragalus* 0.1% + Lonicera 0.1%	28 days	(94)
African catfish, *Clarias gariepinus*	Powdered garlic peel	0.5% incorporated in fish feed	20 days	(51)
Goldfish, *Carassius auratus*	Mixed herbal extracts	400–800 mg/kg diet	4 weeks	(95)
Nile tilapia, *O. niloticus*	Green tea, *Camellia sinensis* L.	0.5 g/kg diet	12 weeks	(96)
Nile tilapia, *O. niloticus*	Cinnamon, *Cinnamomum zeylanicum*	1%/kg diet	8 weeks	(97)
Nile tilapia, *O. niloticus*	American ginseng, *Panax quinquefolium*	1–5 g per kg diet	8 weeks	(98)
Nile tilapia, *O. niloticus*	Mistletoe, *Viscum album coloratum* powder	50 mg per kg diet	80 days	(99)
African catfish, *Clarias gariepinus*	Methanolic extract of *Morus alba* foliage	7 g per kg of feed	30 days	(47)
*O. niloticus* GIFT	Chinese herbal mixture (Astragalus, angelica, hawthorn, licorice and honeysuckle)	0.5–2%/kg diet	4 weeks	(100)
*Labeo rohita*	*Psidium guajava* L. (leaves)	0.5% of diet	60 days	(53)
*L. rohita* fingerling	Tapioca	C/N ratio 15	60 days	(56)
Crucian carp	Polysaccharides of *Ficus carica, Radix isatidis, Schisandra chinensis*	< 0.8/kg diet	4 weeks	(101)
Catfish, *C. gariepinus*	Methanol extract of *Euphorbia hirta* (aerial part)	5 g/kg of fish	30 days	(48)
Silver catfish, *Rhamdia quelen*	*Aloysia triphylla* essential oil	2 ml/kg diet	21 days	(102)
Red hybrid tilapia, *Oreochromis* spp.	Methanolic extract of *Pepperomia pellucida* (leaves)	25 ppm of feed	7 days	(20)
Gibel carp, *Carassius auratus gibelio* var. CAS III	Spirulina, *Arthrospira platensis*	13.53 g/100 g diet	46 days	(55)
Gilthead sea bream, *Sparus aurata*; European sea bass, *Dicentrarchus labrax*	Tetra, *Cotinus coggygria* and mallow, *Malva syvestris*	Tetra 1,000 mg/kg diet and mallow 500 mg/kg diet	60 days	(103)
Common carp, *Cyprinus carpio* L.	*Ginkgo biloba* leaf extract	10 g/kg diet	8 weeks	(49)
*Clarias gariepinus*	Piper beetle, *Psidium guajava, Tithonia diversifolia*	8% per kg of fish feed	28 days	(104)
Nile tilapia, *O. niloticus*	*Pistacia vera-*derived polysaccharide (hull)	5–10 g/kg of feed	60 days	(59)
Common carp, *Cyprinus carpio* L.	Origanum essential oil	15 g/kg diet	8 weeks	(38)
Nile tilapia, *O. niloticus*	Rosemary leaf powder	10 g/kg diet	14 days	(52)
Goldfish, *Carassius auratus*	Chasteberry, *Vitex agnus-castus* extract	15 g/kg of diet	8 weeks	(50)
Goldfish, *Carassius auratus*	Fermented moringa, *Moringa oleifera* Lam	40% replacement of fish meal	50 days	(60)
Grass carp, *Ctenopharyngodon idella*	Curcumin	438.20 mg/kg diet	60 days	(57)
*Pangasianodon hypothalmus*	*Andrographis paniculata*	2% of fish body weight	60 days	(105)
Nile tilapia, *O. niloticus*	Essential oil of thyme, red thyme and pepper rosemary	1.2 mg per g of feed	20 days	(58)
Nile tilapia, *O. niloticus*	Miswak, *Salvadora persica* powder	2% of diet	8 weeks	(29)
Crucian carp	*Ficus carica* polysaccharides	0.4%/kg diet	4 weeks	(106)
Goldfish, *Carassius auratus*	*Flos populi* extract	1-2 g/kg diet	45 days	(92)
Persian sturgeon, *Acipenser persicus*	*Gracilaria persica*	2.5 g/kg diet	8 weeks	(107)

Phytobiotics enhance the fish's immune system by increasing the presence of immune markers such as immunoglobulin M (IgM), nitric oxide and lysozyme. For example, Abdellatief et al. (108) revealed that a combination of sage (*Salvia officinalis*) and *Spirulina platensis* (*Arthrospira platensis*) can boost the immune system of Nile tilapia by increasing their immune response against *Pseudomonas aeruginosa*. Furthermore, phytobiotics, in the form of antioxidant compounds such as phenols and polyphenols, are beneficial for fish health by improving the immune system of aquatic animals against *A. hydrophila* (109). For instance, Naiel et al. (52) revealed that 10 g/kg rosemary (*Salvia rosmarinus*) leaves enhanced Nile tilapia's immune system and increased their disease resistance against *A. hydrophila*. Moreover, the phytobiotic promoted lysozyme and serum catalase activity in fish against MAS.

Overall, phytobiotics with low dose and shorter administration duration are the best candidate as antimicrobial aganet to against MAS. Based on literature survey, phytobiotics such as polysaccharide of *Ficus carica* (106), essential oil of thyme, red thyme and pepper rosemary (58) and methanolic extract of *Pepperomia pellucida* (leaves) (20) were effective in controlling MAS infection as the duration of administration is shorter compared to other phytobiotics. Furthermore, low dose is needed for the mentioned phytobiotics. Further studies need to be carried out in the near future to reveal potential bioactive compounds that present in *Gracilaria persica* (107), *Salvadora persica* (29), *Andrographis paniculata* (105), Curcumin (57), *Moringa oleifera* (60), and many more. The future study findings are important to understand the mechanisms of the bioactive compounds in mitigating *A. hydrophila* impacts on aquatic animals.

## Phytobiotics combined with other antimicrobial agents/therapeutic agents against MAS

The emergence of antibiotic resistance among pathogenic bacteria from aquaculture sites revealed that antibiotics alone are not a sustainable antimicrobial agent (110). Therefore, researchers have proposed alternative solutions, such as phytobiotics. Several studies have also reported on the synergistic effects between phytobiotics and other supplements such as boron (94), probiotics (111, 112), Spirulina (113), and antibiotic (36) to relieve the impacts of *A. hydrophila* in aquaculture species (Table 4). Thus, these combinations are promising antimicrobial agents for cost-effective treatments in aquaculture disease management.

**Table 4:** Medicinal herbs and antimicrobial/therapeutic agents reduce the impacts of *A. hydrophila* on aquatic animals.

**Species**	**Medicinal herbs**	**Supplements**	**Dose**	**Duration**	**References**
Nile tilapia, *O. niloticus*	Chinese medicinal herbs, *Astragalus membranaceus* and *Lonicera japonica*	Boron	*Astragalus membranaceus* 0.1% + 0.05% boron; *Lonicera japonica* 0.1% + 0.05% boron	4 weeks	(94)
Common carp, *Cyprinus carpio* L.	Triherbal extract	Probiotic, Sporolac, *Lactobacillus*	200 mg/kg diet—triherbal extract; 0.1 g/kg diet—probiotic	4 weeks	(112)
Nile tilapia, *O. niloticus*	Garlic powder	*Spirulina platensis* powder	Garlic-−5 g /kg diet Spirulina 10 g/kg diet	8 weeks	(113)
Freshwater white leg shrimp, *Litopenaeus vannamei*	Pomegranate, *Punica granatum* extract (peel)	Florfenicol	0.03 PPM Florfenicol 7.81 PPT *Punica granatum*	7 days	(36)
Nile tilapia, *O. niloticus*	Thyme, Cinnamon	Probiotic, *Bacillus subtilis*	10 g/kg diet—Thyme, Cinnamon; 0.1 g/kg diet—probiotic	8 weeks	(111)

## Conclusion and recommendation

In summary, MAS is a catastrophic disease in the aquaculture industry that can lead to significant economic losses and environmental and public health hazards. Antibiotics have been proven a short-term solution in managing bacterial disease in the aquaculture industry. Therefore, phytobiotics are a viable alternative for aquaculture species health management and for maintaining public health and environmental safety. Despite that, researchers must monitor phytobiotics toxicity as a prerequisite for aquaculture application. Numerous studies have demonstrated the potential of phytobiotics in activating the innate immune system and stimulating disease resistance of aquaculture species against MAS such as essential oil of thyme, red thyme and pepper rosemary whereas methanolic extraction was the best way in preparing phytobiotics. Besides, bioactive compound such as polysaccharide of *Ficus carica* can be effective phytobiotics. Furthermore, applying less ecological footprint antimicrobial agents like phytobiotics can gain consumer confidence in aquaculture products. Nevertheless, there is a gap between the scientific approach and practical use of phytobiotics in aquaculture that can be addressed through knowledge and technology transfer among aquaculturists. Moreover, phytobiotics application in aquaculture remains inconsistent in terms of dosage and duration. These factors are crucial in phytobiotics against MAS because different dosages and duration will lead to variable outcomes. Additionally, phytobiotics efficacy in controlling MAS depends on the source of phytobiotics and environmental conditions. Also, phytobiotics can be administrated orally *via* incorporation in aquaculture feed, thus, a practical, non-stressful, and convenient administration of feed additives for various aquaculture species. Overall, phytobiotics are promising antimicrobial agents in controlling MAS. Most importantly, phytobiotics derived from agricultural wastes have been proven sustainable in aquaculture practices. Further studies need to be carried out in the near future to characterize bioactive compounds that present in phytobiotics and responsible to inhibitory activity against MAS. This is important to understand the mechanisms of the bioactive compounds. In addtion, new potential phytobiotics can be explored from plant families such as Rutaceae, Fabaceae, and Moringaceae.

## Author contributions

ZA: conceptualization, writing—review and editing, and writing—original draft. WW: writing—original draft and writing—review and editing. SM, HC, MH, MI and HV: writing—review and editing. KW: supervision and conceptualization. LS: project administration, writing—original draft, and writing—review and editing. All authors contributed to the article and approved the submitted version.

## Funding

This project was funded by the Ministry of Education, Malaysia, under the Fundamental Research Grant Scheme (FRGS/1/2022/STG03/UMK/03/1), Niche Research Grant Scheme (NRGS) (R/NRGS/A0.700/00387A/006/2014/00152), and Universiti Malaysia Kelantan Matching Grant (Grant no: R/UMK MATCH/A0.700/00387A/008/2022). This research work was partially supported by Chiang Mai University.

## Conflict of interest

The authors declare that the research was conducted in the absence of any commercial or financial relationships that could be construed as a potential conflict of interest.

## Publisher's note

All claims expressed in this article are solely those of the authors and do not necessarily represent those of their affiliated organizations, or those of the publisher, the editors and the reviewers. Any product that may be evaluated in this article, or claim that may be made by its manufacturer, is not guaranteed or endorsed by the publisher.
